# Bacterial Acclimation Inside an Aqueous Battery

**DOI:** 10.1371/journal.pone.0129130

**Published:** 2015-06-12

**Authors:** Dexian Dong, Baoling Chen, P. Chen

**Affiliations:** Department of Chemical Engineering, University of Waterloo, Waterloo, Ontario, Canada; RMIT University, AUSTRALIA

## Abstract

Specific environmental stresses may lead to induced genomic instability in bacteria, generating beneficial mutants and potentially accelerating the breeding of industrial microorganisms. The environmental stresses inside the aqueous battery may be derived from such conditions as ion shuttle, pH gradient, free radical reaction and electric field. In most industrial and medical applications, electric fields and direct currents are used to kill bacteria and yeast. However, the present study focused on increasing bacterial survival inside an operating battery. Using a bacterial acclimation strategy, both *Escherichia coli* and *Bacillus subtilis* were acclimated for 10 battery operation cycles and survived in the battery for over 3 days. The acclimated bacteria changed in cell shape, growth rate and colony color. Further analysis indicated that electrolyte concentration could be one of the major factors determining bacterial survival inside an aqueous battery. The acclimation process significantly improved the viability of both bacteria *E*. *coli *and *B*. *subtilis*. The viability of acclimated strains was not affected under battery cycle conditions of 0.18-0.80 mA cm^-2 ^and 1.4-2.1 V. Bacterial addition within 1.0×10^10^ cells mL^-1^ did not significantly affect battery performance. Because the environmental stress inside the aqueous battery is specific, the use of this battery acclimation strategy may be of great potential for the breeding of industrial microorganisms.

## Introduction

A great deal of research has focused on the use of electric fields [1, 2] and direct currents [3–7] to kill bacteria and yeast for industrial and medical applications. Lethality of an electric field or direct current has been attributed to the electrochemically generated oxidants (including radical oxygen, hydrogen peroxide and ozone), direct oxidation of cellular constituents, and irreversible permeabilization of cell membranes [3, 8].

Many microbial extremophiles (typically bacteria and archaea) can survive in unusual environments such as extreme temperature, pH, pressure, desiccation and salinity [9–10]. However, up to now, there is no report if bacteria could survive in a cycling battery.

Theoretically, bacteria can adapt to new stressful environments by morphological adjustment, genetic mutation, or metabolic alteration [11–13]. The battery system used in this study based on a rechargeable hybrid aqueous battery, consisting of LiMn_2_O_4_ and zinc metal electrodes in an aqueous electrolyte containing 3 M LiCl and 4 M ZnCl_2_, pH adjusted to 4.0 [14]. During battery cycling, hydrogen and oxygen gases were generated [14]. The charge-discharge voltage was between 1.4–2.1 V [14]. Except for significantly higher ionic concentrations in the electrolyte, other conditions inside the battery were not too harsh. Theoretically, it is possible for bacteria to acclimate and consequently survive inside the battery.

The main goal of this research is to evaluate whether bacteria can live inside a battery, where both an electric field and current exist. Bacteria are classified as Gram-negative and Gram-positive types based on structural differences in their cell walls. *Escherichia coli* is a Gram-negative bacterium that grows in a broad pH range of 4.4–10.0, with an optimum pH of 6–7 [15]. *Bacillus subtilis* is a Gram-positive, endospore-forming bacterium with a growth pH range of 4.5–10.0 [16]. Both strains represent typical bacterial characteristics in their types and thus, were chosen for the battery testing in this study.

## Materials and methods

### Battery cell assembly

The electrochemical performance was investigated using two-electrode Swagelok-type cells. Each treatment was tested with at least three repeats. The charge-discharge voltage was between 1.4–2.1 V. Zinc metal foil was used as the anode. The aqueous electrolytes were 0.1 M LiCl and 0.1 M ZnCl_2_ (both Sigma-Aldrich, Canada), and the pH was adjusted to 6.0 using LiOH and HCl. Absorbed Glass Mat (AGM. NSG Corporation) with a thickness of 2.5 mm was used as the separator. The composite cathode consisted of 83 wt % LiMn_2_O_4_ (MTI Co.), 10 wt % acetylene black (Alfa Aesar Co.) and 7 wt % polyvinylidene fluoride (PVDF, Arkema Inc.). N-methyl-2-pyrrolidinone was used as the dispersant (NMP, Sigma, 99.5% purity). The resultant slurry in NMP was coated onto graphite foil (SGL Group Co.). After drying at 70°C in an oven for 24 h, disks of 12 mm in diameter were cut (typical active material load of 2.4 mg cm^-2^) and soaked in the electrolyte solution under vacuum (-0.1 MPa) for 30 min.

### Bacterial culture and acclimation

*E*. *coli* ATCC 11229 and *B*. *subtilis* ATCC 6051 were applied to all battery tests. Bacterial culture and acclimation were always performed with three repeats. One hundred microliters of *E*. *coli* were added into 100 mL LB medium in a 250 mL Erlenmeyer flask. The LB medium was composed of 10 g L^-1^ tryptone, 5 g L^-1^ yeast extract, and 5 g L^-1^ NaCl (pH 7.0–7.5). The culture conditions were 37°C for *E*. *coli* and 30°C for *B*. *subtilis*, 220 rpm, and 15 h unless specified otherwise. For solid plating, 15 g L^-1^ agar powder was added.

After bacteria were cultured for 15 h, 1.5 mL of culture was centrifuged at 5,000 rpm for 2 min. The supernatant was discarded, and the pellet was suspended in 1 mL Milli-Q water. The process was repeated, and the final bacterial pellet was suspended in 500 μL electrolyte containing 0.1 M LiCl, 0.1 M ZnCl_2_ and 2% glucose, pH 6.0. 150 μL of the resulting bacterial suspension was loaded into the AGM separator for battery cycling.

After battery cycling, the battery was disassembled in a bio-safety cabinet. The AGM separator was transferred into a 1.5 mL microfuge tube, and then 1 mL Milli-Q water was added. After vortexing for 2 min, the resulting suspension was transferred into another 1.5 mL microfuge tube. After centrifugation for 2 min at 10,000 rpm, the supernatant was discarded, and the pellet was suspended with 1 mL Milli-Q water, followed by vortexing for 30 sec, and inoculated into 100 mL LB medium for culture. After culturing for less than 48 hours, the medium became cloudy, indicating that bacteria have survived during the battery cycling. However, if the medium remains clear after 48 hours of culturing, then it indicates that the bacteria are dead due to battery cycling. Recovered bacteria were collected and returned to the battery to repeat the above process.

### Scanning electron microscopy (SEM)

After bacteria were cultured for 15 h, 1.0 mL of culture was centrifuged at 5,000 rpm for 2 min and washed twice with 1 mL Milli-Q water. The pellet was collected and suspended in 200 μL Milli-Q water. Eighty microliters of the resulting bacterial suspension was mixed with 80 μL 8% glutaraldehyde and kept at 4°C overnight. 10 μL of the later mixture was dropped onto the silicon wafer and kept for 20 min. The sample was washed with water and wiped with the edge of Kimwipe paper. EtOH at concentrations of 50%, 70%, 90%, and 95% was used to dehydrate the sample. Thirty microliters of EtOH was added to the sample and air dried for approximately 10 min in a bio-safety cabinet. The sample was coated with gold before SEM imaging. The SEM images were taken using a LEO FESEM 1530 field-emission SEM at 5kv.

## Results

### Choices on electrolyte concentration and bacterial medium

**Fig 1:**
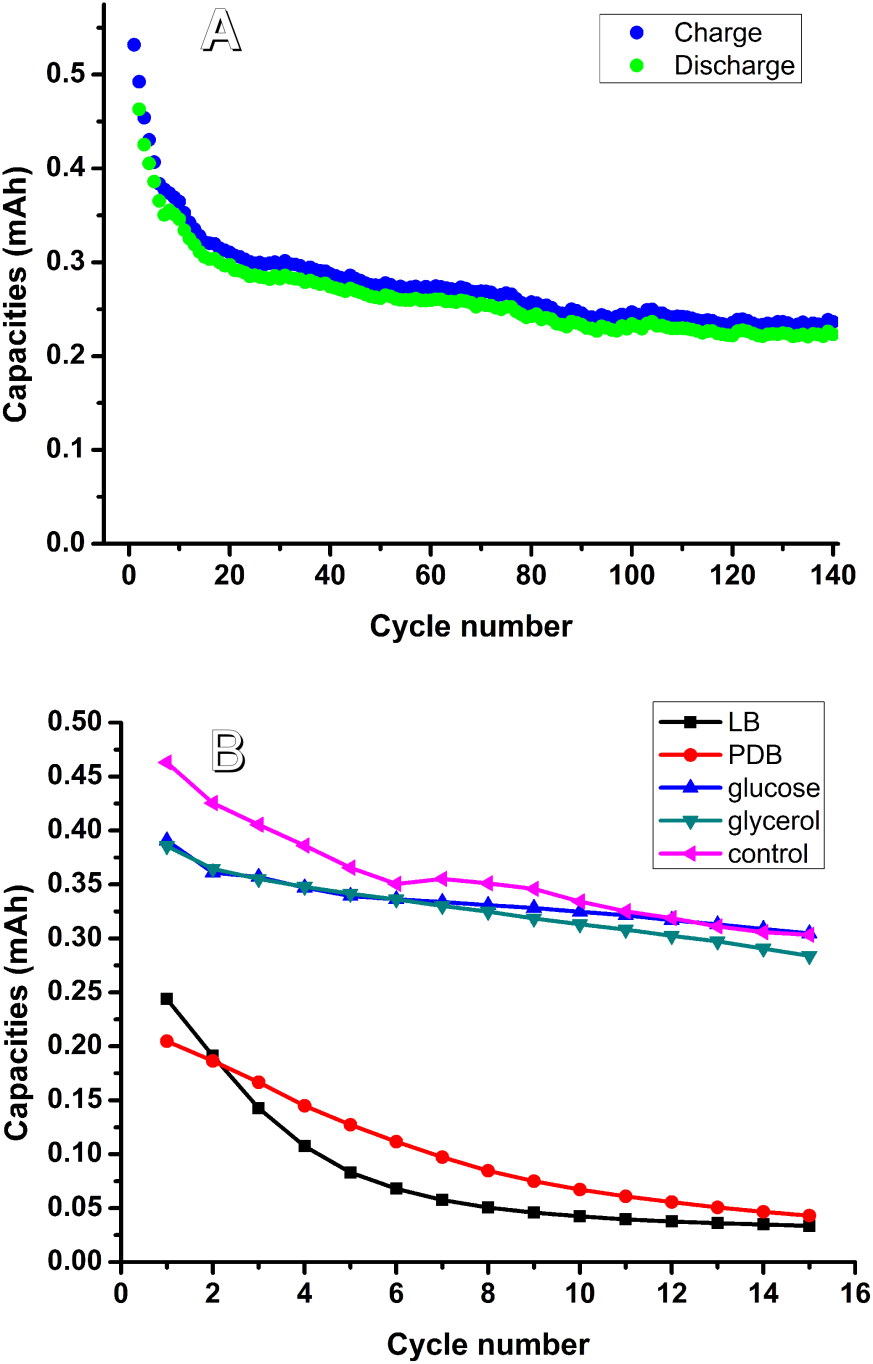
A. battery performance by using 0.1 M LiCl plus 0.1 M ZnCl_2_, pH 6.12 as the electrolyte; B. Effects of different bacterial media on battery cycling.

Common electrolytes for the aqueous battery consist of 3 M LiCl plus 4 M ZnCl_2_, at pH 4.0 or 1 M Li_2_SO_4_ plus 2 M ZnSO_4_, at pH 4.0 [14]. Phosphate buffered saline (PBS) is a buffer solution commonly used in biological research. The total ionic concentration of a PBS solution is approximately 0.15 M, and a common pH used in PBS is approximately 7.0. Unlike PBS, the ionic concentration of an electrolyte in the aqueous battery is much higher, and the pH is too low for bacterial survival. A solution of 0.1 M LiCl plus 0.1 M ZnCl2 (pH 6.12) was chosen as the electrolyte candidate to test battery performance. Using this electrolyte and setting the current density to 0.1 mA cm^-2^ allowed the battery to keep cycling for 648 hours and 140 cycles (Fig 1A). The total capacity gradually decreased from 0.46 to 0.22 mAh. Thus, this electrolyte was chosen as the basic electrolyte for further tests.

Regarding the media used, 10% LB medium or 10% Potato Dextrose Broth (PDB) was added to the electrolyte. Results showed that battery capacity faded quickly after the addition of LB or PDB (Fig 1B). Addition of M9 medium caused high precipitation in the electrolyte. Upon addition of 2% glucose or 2% glycerol, the battery performance was similar to that without any media added (control) (Fig 1B). It can be concluded that both multi-component organic and inorganic media are not suitable for the battery with living bacteria. Two percent glucose was chosen as the medium for the battery testing.

### Bacterial acclimation

After bacteria (approximately 1.0×10^10^ cells) were put into the electrolyte and the battery was cycled for 12, 24 or 48 hours, the bacteria in the electrolyte were taken out for LB culture. If the culture became cloudy, it demonstrated that the bacteria could propagate after cycling and could be designated as viable. Based on this approach, it was observed that the original *E*. *coli* strain was viable after 24 hours battery cycling but dead after 48 hours battery cycling while the original *B*. *subtilis* strain was viable after 12 hours battery cycling but dead after 24 hours battery cycling. Based on the theory of bacterial acclimation, bacteria could adapt themselves to a new stressful environment by mutation or by alteration of their metabolism. Both bacteria were subjected to 10 cycles of acclimation. This procedure was performed by repeatedly cycling the battery for 24 hours followed by bacterial culturing (for the first 3 cycles, *B*. *subtilis* was acclimated by 12 hour battery cycling then bacterial culturing). After 10 cycles of acclimation, *E*. *coli* and *B*. *subtilis* remained viable after 72 hours and 96 hours of cycling, respectively.

### Bacterial characteristics

**Fig 2:**
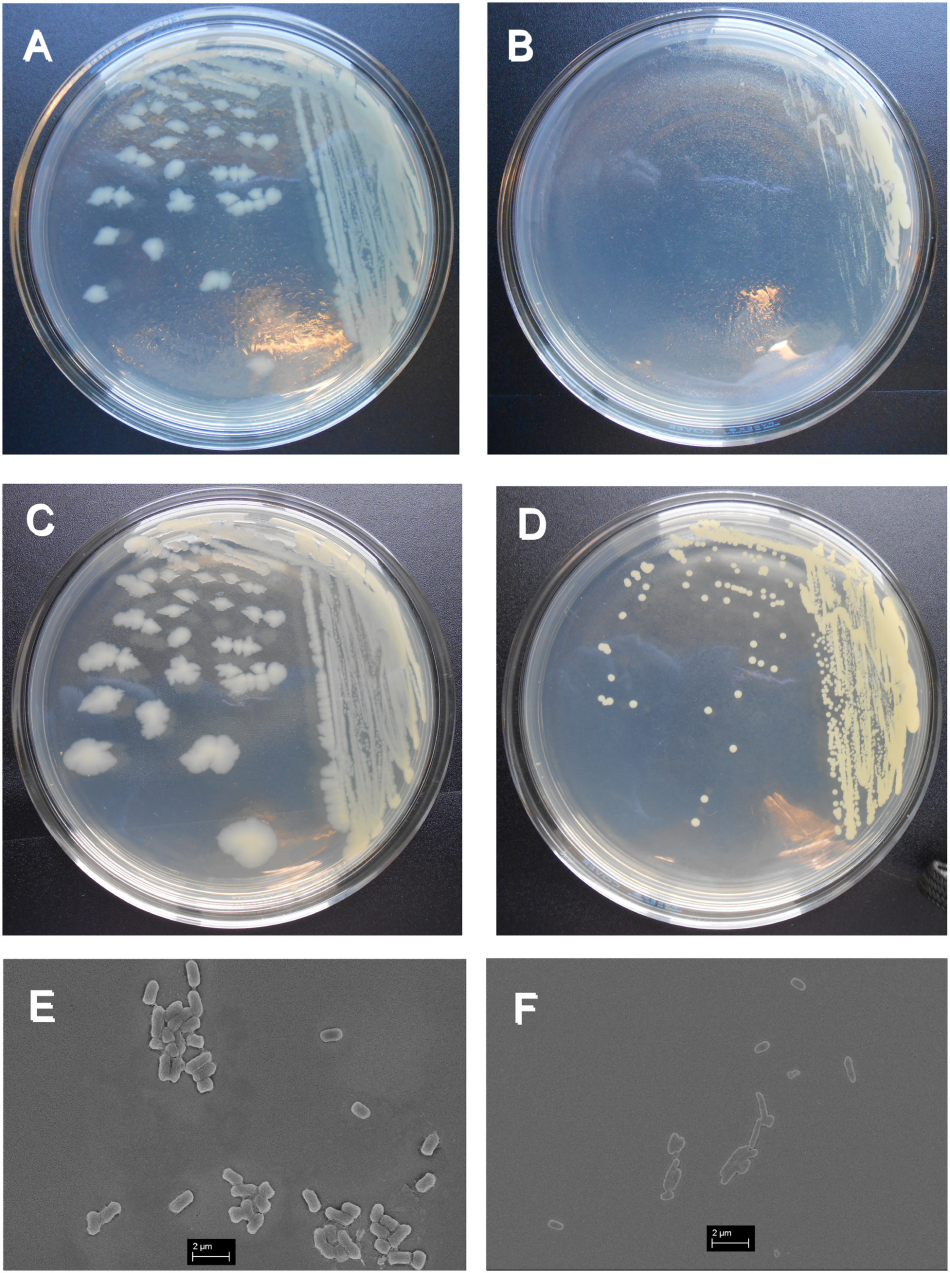
SEM images and Colony growths of original *E*. *coli* and acclimated *E*. *coli*. A: 24 h growth of original *E*. *coli;* B: 24 h growth of acclimated *E*. *coli;* C: 44 h growth of original *E*. *coli;* D: 44 h growth of acclimated *E*. *coli;* E: SEM image of original *E*. *coli;* F: SEM image of acclimated *E*. *coli*.

Fig 2 shows the growth and morphology of both the original and acclimated *E*. *coli*. A significant change was that, after centrifugation, the pellet color of the acclimated *E*. *coli* was yellow. After growth on LB plates for 24 h, the original *E*. *coli* formed a clear, larger colony, and the colony surface appeared slightly whitish and translucent (Fig 2A). The acclimated *E*. *coli* grew slowly, and colonies were not visible after 24 hours (Fig 2B). After 44 h growth on plates, the acclimated *E*. *coli* colonies appeared as small, yellowish circular spots (Fig 2D) while the original *E*. *coli* was much bigger (Fig 2C). These results demonstrated that the growth rate of the acclimated *E*. *coli* was lower than that of the original *E*. *coli*. According to the SEM images, the original *E coli* were rod-shaped, and each bacterium measured approximately 0.5 μm in diameter by 0.5–1.0 μm in length (Fig 2E). The acclimated *E*. *coli* became egg-shaped; the diameter of the bacterium became smaller (approximately 0.25 μm), and the length of the bacterium varied within the regular range (0.5–2 μm in length) for *E*. *coli* (Fig 2F).

**Fig 3:**
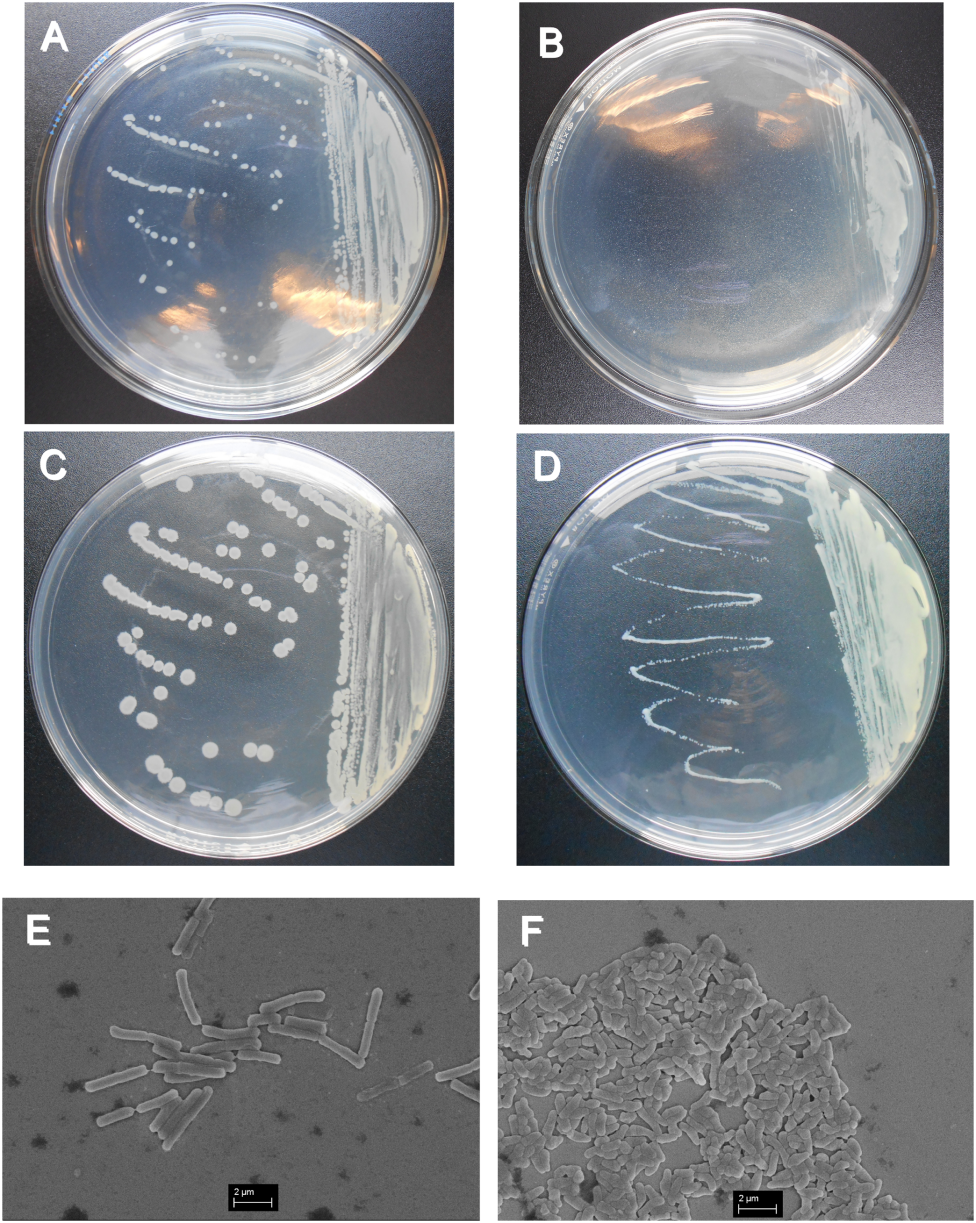
SEM images and Colony growths of original *B*. *subtilis* and acclimated *B*. *subtilis*. A: 24 h growth of original *B*. *subtilis;* B: 24 h growth of acclimated *B*. *subtilis;* C: 48 h growth of original *B*. *subtilis;* D: 48 h growth of acclimated *B*. *subtilis;* E: SEM image of original *B*. *subtilis;* F: SEM image of acclimated *B*. *subtilis*.

For *B*. *subtilis*, the growth rate of the acclimated *B*. *subtilis* (Fig 3B and 3D) also decreased compared to the original *B*. *subtilis* (Fig 3A and 3C). The colony color of the acclimated *B*. *subtilis* changed to light yellow (Fig 3D). According to the SEM images, both the original and acclimated *B*. *subtilis* were rod-shaped, and the diameter of the bacterium was approximately 0.25 μm. For the length of the bacterium, the acclimated *B*. *subtilis* became shorter (1 μm compared to 2–4 μm). The shortest cells (0.5 μm shown in Fig 3F) might be the endospores of *B*. *subtilis*, which are usually produced under harsh conditions. In addition, the acclimated *B*. *subtilis* did not grow at 37°C while the original *B*. *subtilis* did.

### Effect of electrolyte concentration on bacterial viability

**Fig 4:**
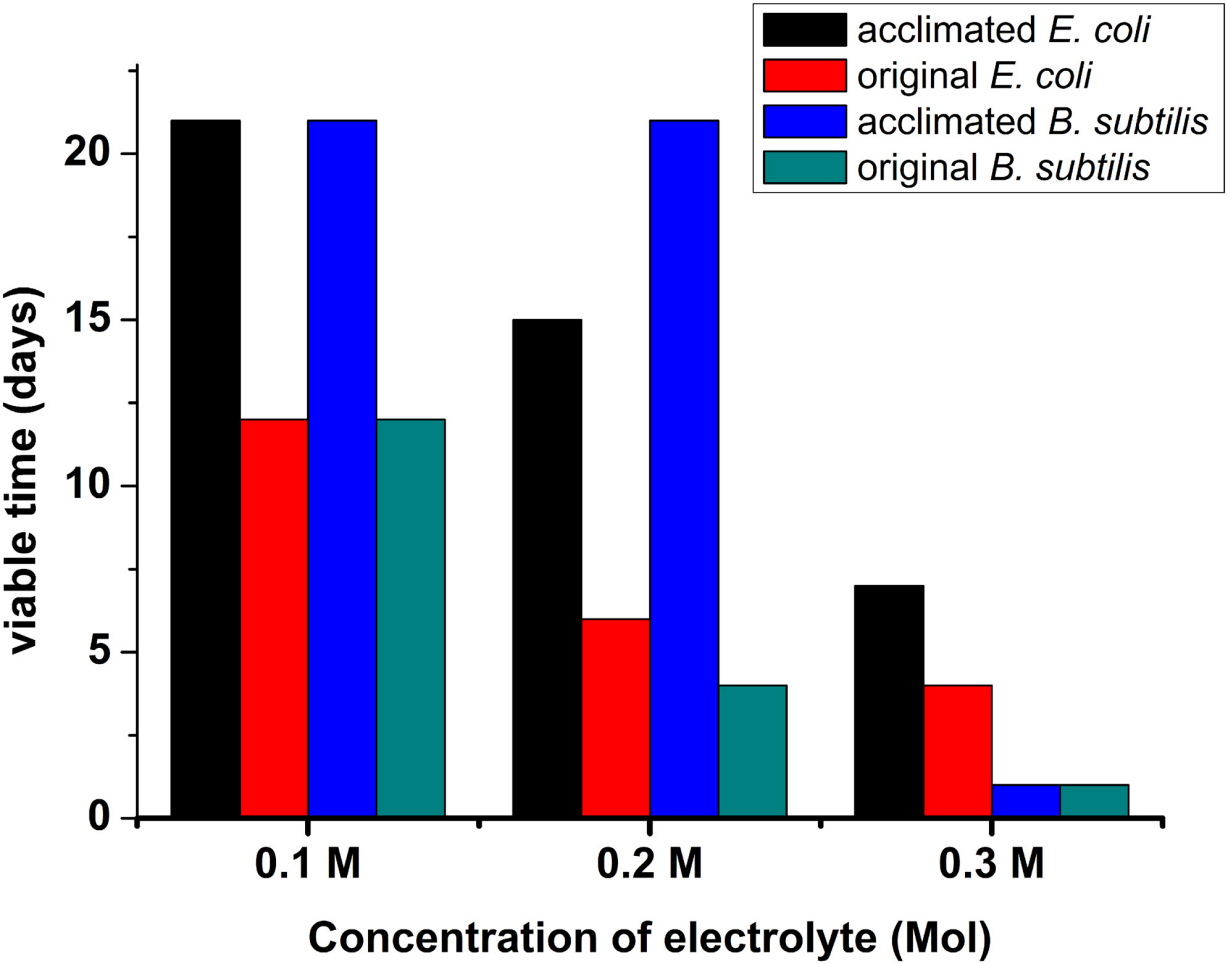
Effect of electrolyte concentration on the viability of acclimated *E*. *coli* and *B*. *subtilis*.

Electrolyte concentration is one of the major factors for bacterial survival inside a battery. The original and acclimated *E*. *coli* and *B*. *subtilis* strains (with some LB media) were put into 0.1 M, 0.2 M and 0.3 M electrolytes for several days and then removed for LB culturing. The results are shown in Fig 4. The original *B*. *subtilis* lost viability within 1 day in the 0.3 M electrolyte, 4 days in the 0.2 M electrolyte and 12 days in the 0.1 M electrolyte. The original *E*. *coli* lost viability within 4 days in the 0.3 M electrolyte, 6 days in the 0.2 M electrolyte, and 12 days in the 0.1 M electrolyte. After 10-cycle acclimation, viability of both *E*. *coli* and *B*. *subtilis* significantly improved. Both acclimated *E*. *coli* and *B*. *subtilis* survived for 21 days in the 0.1 M electrolyte. For the 0.2 M electrolyte, the acclimated *E*. *coli* survived for 15 days, but the acclimated *B*. *subtilis* survived for 21 days. For the 0.3 M electrolyte, acclimated *E*. *coli* survived for only 7 days, and *B*. *subtilis* survived less than one day.

### Effect of current density on bacterial viability

Because the acclimated *E*. *coli* survived for 7 days in the 0.3 M electrolyte, 0.1 M, 0.2 M, 0.3 M electrolytes were chosen to test the effect of current density. For the 0.1 M electrolyte, current densities were set from 0.18 to 0.53 mA cm^-2^. For the 0.2 M electrolyte, current densities were set from 0.27 to 0.62 mA cm^-2^. For the 0.3 M electrolyte, current densities were set from 0.53 to 0.88 mA cm^-2^. After 72 h cycling, all of the bacteria were removed from the electrolyte and cultured in LB medium. The results showed that all of the bacteria from these treatments were viable. It can be concluded that the viability of the acclimated *E*. *coli* is not affected by the cycle conditions of 0.18–0.88 mA cm^-2^ and 1.4–2.1 V.

Based on the results showing that the acclimated *B*. *subtilis* could not survive over 1 day in the 0.3 M electrolyte, 0.1 M and 0.2 M electrolytes were chosen for further tests. For the 0.1 M electrolyte, current densities were set from 0.18 to 0.53 mA cm^-2^. For the 0.2 M electrolyte, current densities were set from 0.35 to 0.80 mA cm^-2^. After 72 h cycling, results showed that all of the bacteria from these treatments were viable. It can be concluded that the viability of the acclimated *B*. *subtilis* is not affected by the cycle conditions of 0.18–0.80 mA cm^-2^ and 1.4–2.1 V.

### Effect of bacterial addition on battery performance

**Fig 5:**
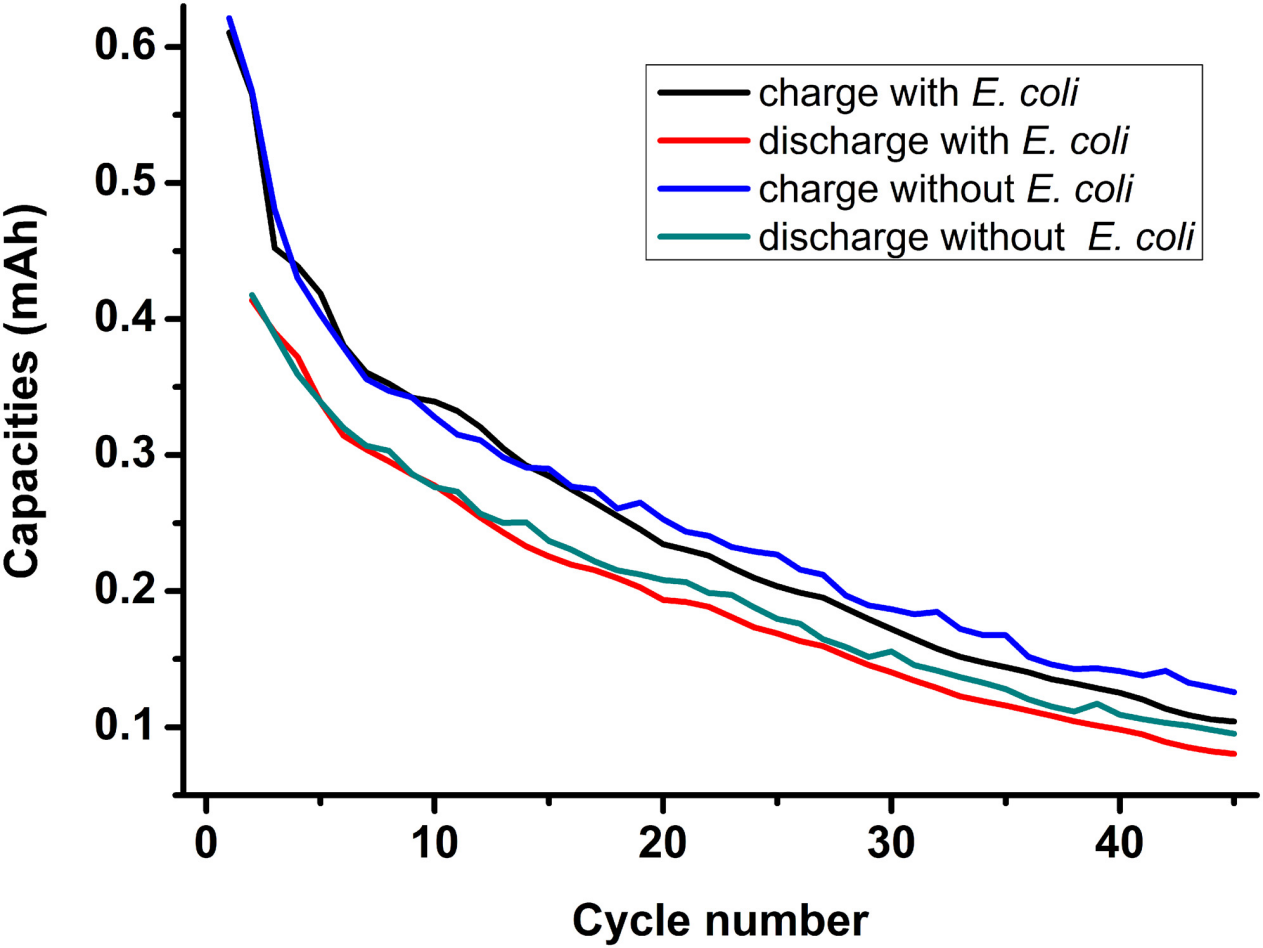
Effect of bacteria addition on battery performance.

Addition of bacteria decreased the electrolyte conductivity. However, this reduction was not significant. For the 0.2 M electrolyte, adding 1.0×10^10^ cells mL^-1^ of *E*. *coli* decreased the electrolyte conductivity from 42.4 mS ± 0.1 cm^-1^ to 42.2 mS ± 0.1 cm^-1^(data from six repeats). After the bacterial suspension was dropped onto the surface of the AGM (Absorptive Glass Mat) separator, the bacteria condensed on the surface. Facing this bacteria-condensed side of the separator on the zinc anode, the battery could not be charged to 2.1 V. Facing the bacteria-condensed side of the separator on the LMO (LiMn_2_O_4_) cathode, the battery could cycle but the first charge curve was not as smooth as the one without bacterial addition. There was a hump in the beginning of the first charge curve. This hump indicated that bacteria contacting the LMO cathode could negatively affect the cathode function. It demonstrates that bacteria cannot be in contact with both electrodes. After the AGM separator was torn in two and the bacterial suspension was dropped in the middle of the separator, the negative hump effect disappeared. Fig 5 shows the comparison of battery performance with and without *E*. *coli* addition (0.1 M electrolyte and 0.27 mA cm^-2^ current density). According to the charge curves and discharge curves shown in Fig 5, the differences caused by bacterial addition are insignificant, thus implying that bacterial addition under 1.0×10^10^ cells mL^-1^ does not significantly affect battery performance.

## Discussion

### Analysis of environmental stress inside the aqueous battery

**Fig 6:**
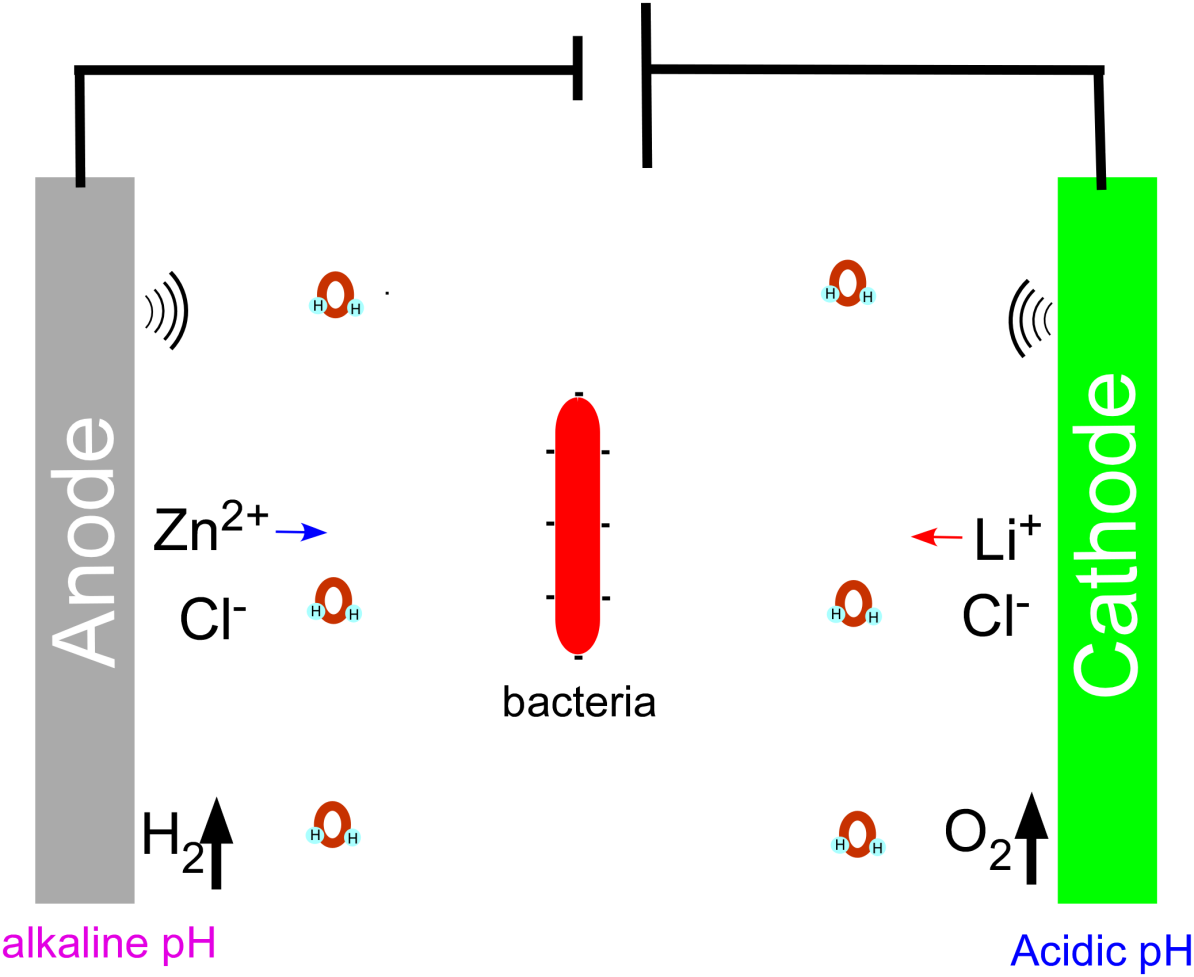
Schematic diagram for the environmental stress inside aqueous battery.

The environmental stress encountered by the bacteria placed inside our aqueous battery is specific (Fig 6). It includes (1) Ion shuttle: During charge, Li^+^ ions deintercalate from the LMO cathode, hydrate with water [17] and move to the anode. Meanwhile, hydrated Zn^2+^ ions in the electrolyte [18] move to the anode, obtain electrons, become Zn atoms and are deposited on the surface of the anode. During discharge, Zn atoms lose electrons, become Zn^2+^, hydrate into the electrolyte and move to the LMO cathode. Hydrated Li^+^ ions move to the cathode, obtain electrons and intercalate into the LMO matrix. Cl^-^ ions also move together with cations during both charge and discharge. (2) pH gradient: Due to the ion shuttling, a large pH gradient inside the battery generates when the electricity is charged. Sandvik et al [7] measured the pH with pH strips while the current was running and found that after five minutes, pH gradients were approximately 3 at the anode, 9 at the cathode and between 6 and 7 at the center of the wells in their experimental system. Any pH gradient across the well would be expected to rapidly dissipate when the current was stopped due to diffusive and convective mixing, and there was no net change in the pH of the solution. The aqueous battery also belongs to the non-buffered stationary system. The pH value at different locations of the electrolyte should be different and would vary during charge and discharge phases. A potentially large pH change would occur in the localized vicinity of both LMO cathode and Zn anode. (3) Water electrolysis: The electrolyte in the aqueous battery contains water molecules. Water electrolysis could generate a variety of chemical oxidants [3, 5] such as radical oxygen and radical hydrogen, which could oxidize or reduce all of the components inside the battery. Such oxidants could be one of the reasons for the inactivation and lethality of the added bacterial cells. (4) Gas generation: Due to the water electrolysis, radical oxygen interacts and forms gaseous oxygen, and similarly, radical hydrogen forms gaseous hydrogen. Radical oxygen oxidizes the carbon contained in the LMO cathode or graphite current collector to form gaseous carbon dioxide. (5) Electric field: When direct current is applied to a pair of electrodes, an electric field inside the battery is generated. It induces a transmembrane potential inside the bacteria placed in the electrolyte, thus changing bacterial cell shape and surface hydrophobicity, the orientation of membrane lipids, net surface charge, DNA and protein synthesis, and bacterial activity and metabolism [19].

### Further improvement of battery acclimation

The survival mechanism for both acclimated bacterial strains is unclear. Theoretically, any microorganism living in a high-salt environment must balance its cytoplasm osmotically with its environmental medium. There are two fundamentally different strategies to achieve osmotic balance: “Salt-In” and “Low Salt-In” [20]. Further research is required to understand which strategy was adopted in this study.

The AGM separator used is highly porous, and its pore size (~4–45 μm) [21] is large enough to let bacteria move freely inside a battery. During battery cycling, most of the bacteria, usually carrying a negative charge on the surface, move to both electrodes and are killed. The lower rate of bacterial propagation observed during acclimation suggested this killing occurred. The possible reasons could be the significantly elevated pH and the action of the strong electric field in the localized vicinity of both electrodes [4]. Using cell entrapment techniques [22] could avoid this problem. However, the effects of these techniques on bacterial acclimation need to be further studied.

### Potential application of battery acclimation

According to the data mentioned above, following acclimation, both *E*. *coli* and *B*. *subtilis* changed in cell shape, growth rate and colony color. This result indicates that the special environmental stress inside the battery induced the mutation or metabolic alteration of both bacterial strains. This battery acclimation strategy has great potential to be used for the breeding of industrial microorganisms. How the environmental stress inside the aqueous battery influences biological systems and whether it causes any adverse effect on microorganisms are questions that require further investigation. In addition, viruses, fungi, plant seeds or stem cells can be placed inside the aqueous battery to test the influence of the battery environment on biological systems. The outcome of further research will significantly enrich the knowledge of battery acclimation.

## Conclusions

In this study, at room temperature with an electrolyte of 0.1 M LiCl plus 0.1 M ZnCl2 at pH 6.0 and a medium of 2% glucose, both *E*. *coli* and *B*. *subtilis* were subjected to 10 cycles of battery acclimation under voltages of 1.4 and 2.1 V at a constant current density of 0.25 mA cm^-2^. The results showed that both *E*. *coli* and *B*. *subtilis* survived in the operating battery for over 3 days. The acclimated *E*. *coli* became smaller, egg-shaped, and yellowish. The acclimated *B*. *subtilis* became shorter and light yellow. Both the acclimated bacteria showed slower growth rates compared to the original strains. Further studies indicated that electrolyte concentration could be one of the major factors for bacterial survival inside the aqueous battery. Battery acclimation significantly improved the viability of both bacteria *E*. *coli* and *B*. *subtilis*. The viability of the acclimated strains was not affected under the battery cycle conditions of 0.18–0.80 mA cm^-2^ and 1.4–2.1 V. Bacterial addition within 1.0×10^10^ cells mL^-1^ did not significantly affect battery performance. The environmental stresses inside the aqueous battery include ion shuttle, pH gradient, water electrolysis, gas generation and electric field. The combination of these stresses is specific and can induce some genetic mutation or metabolic alteration of bacteria. Therefore, this battery acclimation strategy holds great promise for the breeding of functionalized microorganisms for industrial and medical applications.
